# Hydration number: crucial role in nuclear magnetic relaxivity of Gd(III) chelate-based nanoparticles

**DOI:** 10.1038/s41598-017-14409-6

**Published:** 2017-10-25

**Authors:** Rustem Zairov, Gulshat Khakimullina, Sergey Podyachev, Irek Nizameev, Georgy Safiullin, Rustem Amirov, Alberto Vomiero, Asiya Mustafina

**Affiliations:** 1 A. E. Arbuzov Institute of Organic and Physical Chemistry, Kazan Scientific Center of Russian Academy of Sciences, Arbuzov str., 8, 420088 Kazan, Russia; 2 Kazan (Volga region) Federal university, Kremlyovskaya str., 18, 420008 Kazan, Russia; 3 Kazan National Research Technical University, K. Marx str., 10, 420211 Kazan, Russia; 4 Zavoisky Physical -Technical Institute of Kazan Scientific Center of Russian Academy of Sciences, Sibirsky tract, 10/7, 420029 Kazan, Russia; 5 Division of Materials Science, Department of Engineering Sciences and Mathematics, Luleå University of Technology, SE-971 87 Luleå, Sweden

## Abstract

Today, nanostructure-based contrast agents (CA) are emerging in the field of magnetic resonance imaging (MRI). Their sensitivity is reported as greatly improved in comparison to commercially used chelate-based ones. The present work is aimed at revealing the factors governing the efficiency of longitudinal magnetic relaxivity (r_1_) in aqueous colloids of core-shell Gd(III)-based nanoparticles. We report for the first time on hydration number (q) of gadolinium(III) as a substantial factor in controlling r_1_ values of polyelectrolyte-stabilized nanoparticles built from water insoluble complexes of Gd(III). The use of specific complex structure enables to reveal the impact of the inner-sphere hydration number on both r_1_ values for the Gd(III)-based nanoparticles and the photophysical properties of their luminescent Tb(III) and Eu(III) counterparts. The low hydration of TTA-based Gd(III) complexes (q ≈ 1) agrees well with the poor relaxivity values (r_1_ = 2.82 mM^−1^s^−1^ and r_2_ = 3.95 mM^−1^s^−1^), while these values tend to increase substantially (r_1_ = 12.41 mM^−1^s^−1^, r_2_ = 14.36 mM^−1^s^−1^) for aqueous Gd(III)-based colloids, when macrocyclic 1,3-diketonate is applied as the ligand (q ≈ 3). The regularities obtained in this work are fundamental in understanding the efficiency of MRI probes in the fast growing field of nanoparticulate contrast agents.

## Introduction

Magnetic resonance imaging (MRI) has become an indispensable tool in cancer diagnostics due to its outstanding ability to differentiate soft tissues. Paramagnetic metal ions (Gd^3+^, Mn^2+^) have been widely used in MRI medical practice as contrast enhancing agents (CAs) in the form of low-molecular chelates^1,2^. The use of CAs improves greatly the accuracy of diagnosis in terms of higher specificity and better tissue characterization via active/passive tumor targeting and sufficient reduction of the relaxation times of water protons. Formerly, the greatest progress in MRI was achieved by applying low-molecular Gd(III) chelates, resulted in approval from the Food and Drug Administration (FDA) of series MRI CAs for medical usage ([Gd(dtpa)(H_2_O)]^2−^ (Magnevist: BSP), ([Gd(dota)(H_2_O)]^−^ (Dotarem: Guerbet)^3^, etc. Decades of extensive use of Gd-based molecular CAs revealed significant drawbacks, related to toxicity of Gd-deposits in tissues^4,5^. Strategies to minimize negative aftereffects of gadolinium usage have been developed recently. The decrease of gadolinium dosage via improvement of CA sensitivity, as well as the enhancement of thermodynamic stability of the corresponding chelates is stated as the most promising way in this direction^6–8^. To increase the sensitivity of CA, the rotational time of Gd-containing low-molecular chelate was found to be a fundamental factor. Rotational kinetics was shown to be slowed down via encapsulation of small Gd-chelates into nanosized aggregates. Literature data demonstrate several most efficient synthetic approaches of such encapsulation resulting in considerable enhancement of relaxivity^9–15^. Non-covalent entrapping of Gd-chelates by macromolecules^9,10^ or their interaction with proteins^11,12^ can be regarded as the self-sufficient approaches. Inclusion of Gd-chelates into hard nanoparticles is represented in literature by plenty of works due to its efficiency in slowing down rotational kinetics of Gd-chelates^13–21^. Transformation of non-covalently encapsulated Gd-chelates into hydrogels^13^ or their silica coating^14–17^ represents very efficient synthetic approaches of making efficient MR contrast agents, although more simple synthetic approach based on precipitation of ultra-small water insoluble Gd-based nanoparticles has been gained great attention in recent time^18–21^.

Relaxivity is also directly proportional to the number of water molecules coordinated to paramagnetic label and residency time of water molecules^16^. Since increase in hydration number (q) results in relaxivity boost, to create a Gd-based MRI label with high q without considerable loss in kinetic and thermodynamic stability of gadolinium(III) complex is very challenging.

The greatest progress in relaxation parameters observed recently is related to the huge number of studies involving gadolinium-doped nanoparticles of different types^13–21^. High chemical stability, large Gd(III) payload (hence, high relaxivity per particle), target specificity and multimodality are the main factors determining the superiority of nanoparticulate CAs compared to the commercial low-molecular ones. An inner-sphere water molecules exchange and a size of hard Gd(III)-based nanoparticles are highlighted as key factors affecting the relaxivity of aqueous Gd(III)-based colloids^22–25^. However, high relaxation efficiency of nanoparticulate MRI probes has been combined with the understanding of the key driving factors only on a qualitative level, since no theoretical framework is known for accurate interpretation of relaxivities in Gd(III)-based colloids.

Herein, we aim to correlate MRI relaxation efficiency of aqueous colloids based on Gd(III) chelates with hydration number of gadolinium. We developed Gd-based colloids on a platform of two complexes, namely [Gd(TTA)_3_·**1**], where TTA^−^ is thenoyltrifluoroacetonate, **1** is phosphine oxide derivative, and Na[Gd·**2**], where **2** is tetra-1,3-diketone calix[4]arene with different hydration numbers and give here the comparative analysis of their colloidal and magnetic relaxation characteristics. Our findings reveal the hydration number of Gd(III) as a determining factor in tuning the MRI contrasting ability of nanoparticulate contrast agents. This is of great importance in designing new tomography labels with advanced characteristics.

## Results and Discussion

To evaluate correctly the role of the number of coordinated water molecules in nuclear magnetic relaxivity efficiency of gadolinium(III)-based colloids it is of great importance to choose proper Gd(III) complexes. Similar ligand environment and different number of water molecules in the first coordination sphere of paramagnetic cation and, since we are dealing with colloids, similar shape and size of the nanoparticles are the main requirements in this respect.

**Figure 1:**
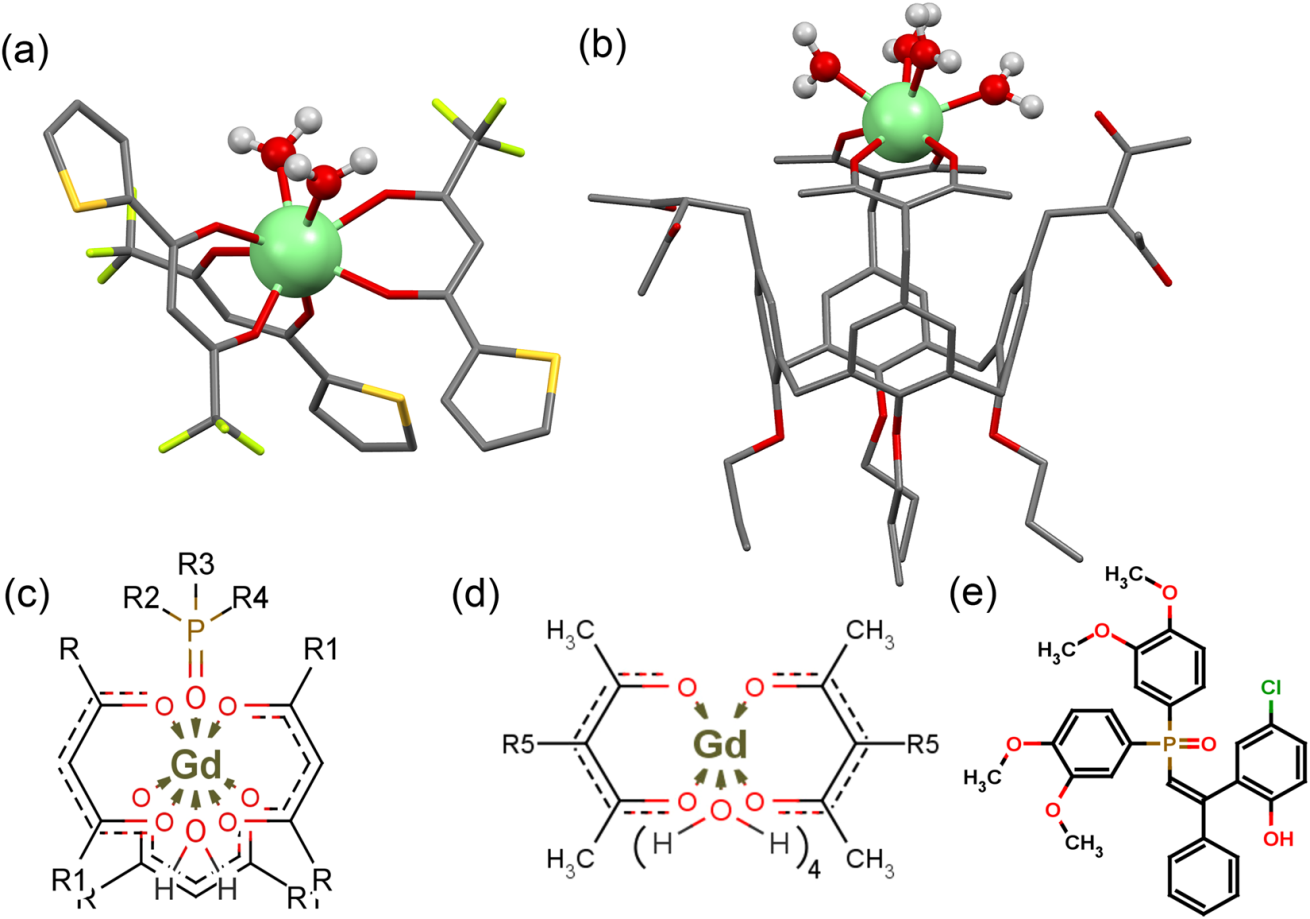
Structure of [Gd(TTA)_3_·2H_2_O] (**a**)^26^ and of Na[Gd·**2**·4H_2_O] (**b**)^27^ (SPARKLE/PM7 optimi*zation*). Schematic illustration of coordination environment of gadolinium(III) in [Gd(TTA)_3_·**1**·H_2_O] (**c**) and Na[Gd·**2** *·*4H_2_O] (**d**) complexes. Structure of *phosphine oxide* 1 (**e**). Inner sphere water molecules are omitted in the text for clarity.

[Gd(TTA)_3_·**1**] (Fig. 1a) ^28^ and Na[Gd·**2**] (Fig. 1b) ^27^ complexes with different hydration numbers were chosen as the basis of the synthesis of the PSS-stabilized aqueous colloids. The hydration number (q) for [Gd(TTA)_3_·**1**] is assumed to be one. This assumption is based on the documented X-ray structure for [Eu(TTA)_3_∙2H_2_O]^26^, while the inner-sphere coordination of phosphine oxide **1** to [Eu(TTA)_3_∙2H_2_O] previously reported for the complexes in organic solutions and in the corresponding PSS-stabilized colloids^28^ should expel at least one water molecule from the inner-sphere in accordance with Fig. 1c.

The macrocyclic calix[4]arene platform (Fig. 1b), being covalently linked to 1,3-diketonates and not contributing to first coordination sphere of Gd(III) directly in ligand 2, plays substantial role in ligand to metal coordination mode. It was previously proven that only two of the four 1,3-diketonate groups of 2 coordinate to Gd(III) cation, forming bis-chelated coordination mode^27^, while the coordination of other two 1,3-diketone groups is sterically hindered. Taking into account that coordination number of Gd(III) is eight^29^, four inner-sphere water molecules are anticipated for Gd(III)-centers in Na[Gd·**2**]-based aqueous colloids. Nevertheless, these assumptions should be confirmed by the experimental data. Moreover, the similarity in shape and size of these lower and higher hydrated colloids is another important prerequisite for revealing the hydration effect on photophysical or magnetic relaxation parameters of the colloids. For these reasons atomic force microscopy (AFM) and transmission electron microscopy (TEM) studies were performed for the colloids.

### Colloidal properties of Gd(III) chelate-based nanoparticles

The synthesis of hydrophilic colloids based on [Gd(TTA)_3_·**1**] and Na[Gd·**2**] complexes coated with PSS anions was first developed for [Eu(TTA)_3_·**1**]^30^. The developed synthetic method is simple and uniform, which enables to extend it to various complexes of different lanthanides(III)^31,32^. It is worth noting that a water insolubility is the main prerequisite for the conversion of the lanthanide complexes into PSS-stabilized nanoparticles, since the latter are formed under dropwise addition of lanthanide complex organic solution to aqueous solution of PSS at 0.5 M of sodium chloride under effective stirring (for detailed synthetic procedure see supplementary information, SI). Adsorption of PSS polyanions on the surface of the precipitated nanosized complex species makes a hydrophilic exterior layer, which is a stabilizing factor in terms of colloidal stability of the PSS-stabilized nanoparticles. The electrokinetic potential measurements confirm the negative exterior charge of the colloids (Table 1SI).

**Figure 2:**
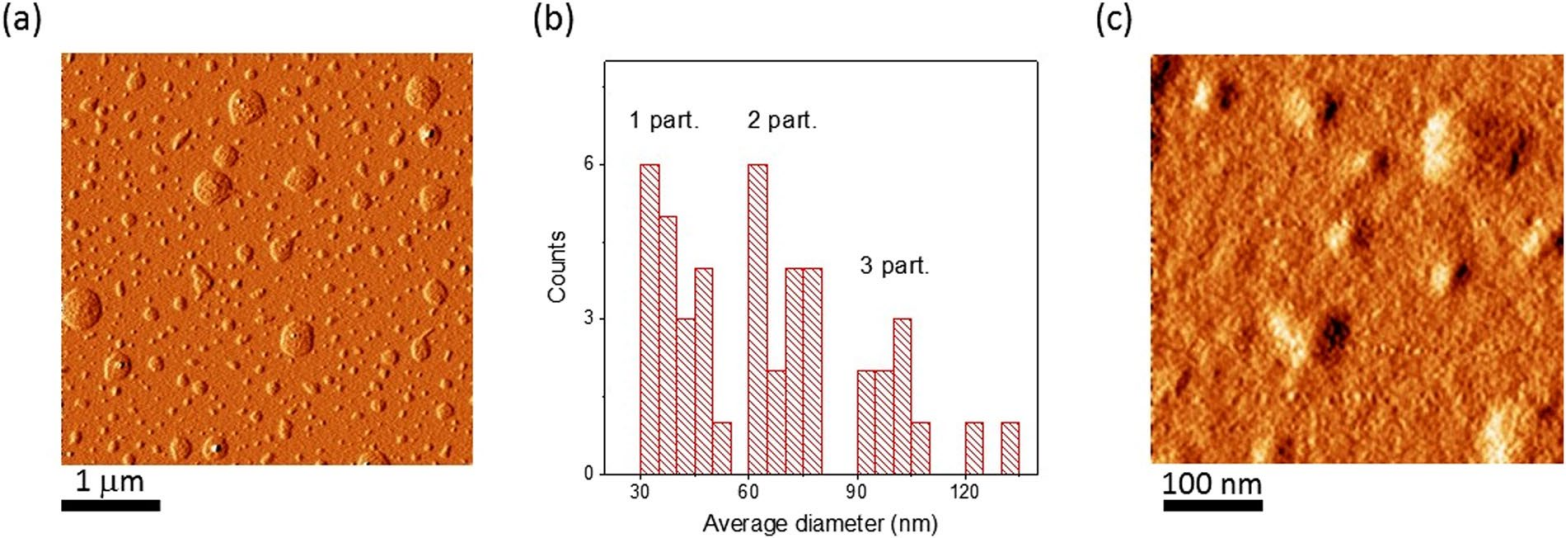
AFM images of dried PSS-coated [Gd(TTA)_3_·**1**] colloids at different magnification (**a**,**c**) and average diameter distribution analysis (**b**).

Atomic force microscopy is a powerful tool in analysis of size, shape and aggregation of nanoparticles in the dried state. Figure 2 introduces AFM images of the colloids based on [Gd(TTA)_3_·**1**]. Multiple spherical particles of different diameters are detected through AFM performed at low magnification (Fig. 2a). These species are 30-45 nm sized single particles of PSS-[Gd(TTA)_3_·**1**] as well as their di-, tri- and polyagglomerates, as it is evident from the diameter distribution analysis (Fig. 2b) and AFM images at higher magnification (Fig. 2c). DLS measurements in Gd(III)-based colloidal systems reveal 150–170 nm sized species existing in aqueous solution (Table 1SI). The significant difference in particle diameters between electron microscopy/AFM and DLS may be explained by the hydration-mediated swelling effect and the difference in the measured parameter. In contrast to AFM and TEM, DLS technique defines the hydrodynamic diameter, reflecting the mean size of the species migrating in aqueous solution, including counter-ions.

**Figure 3:**
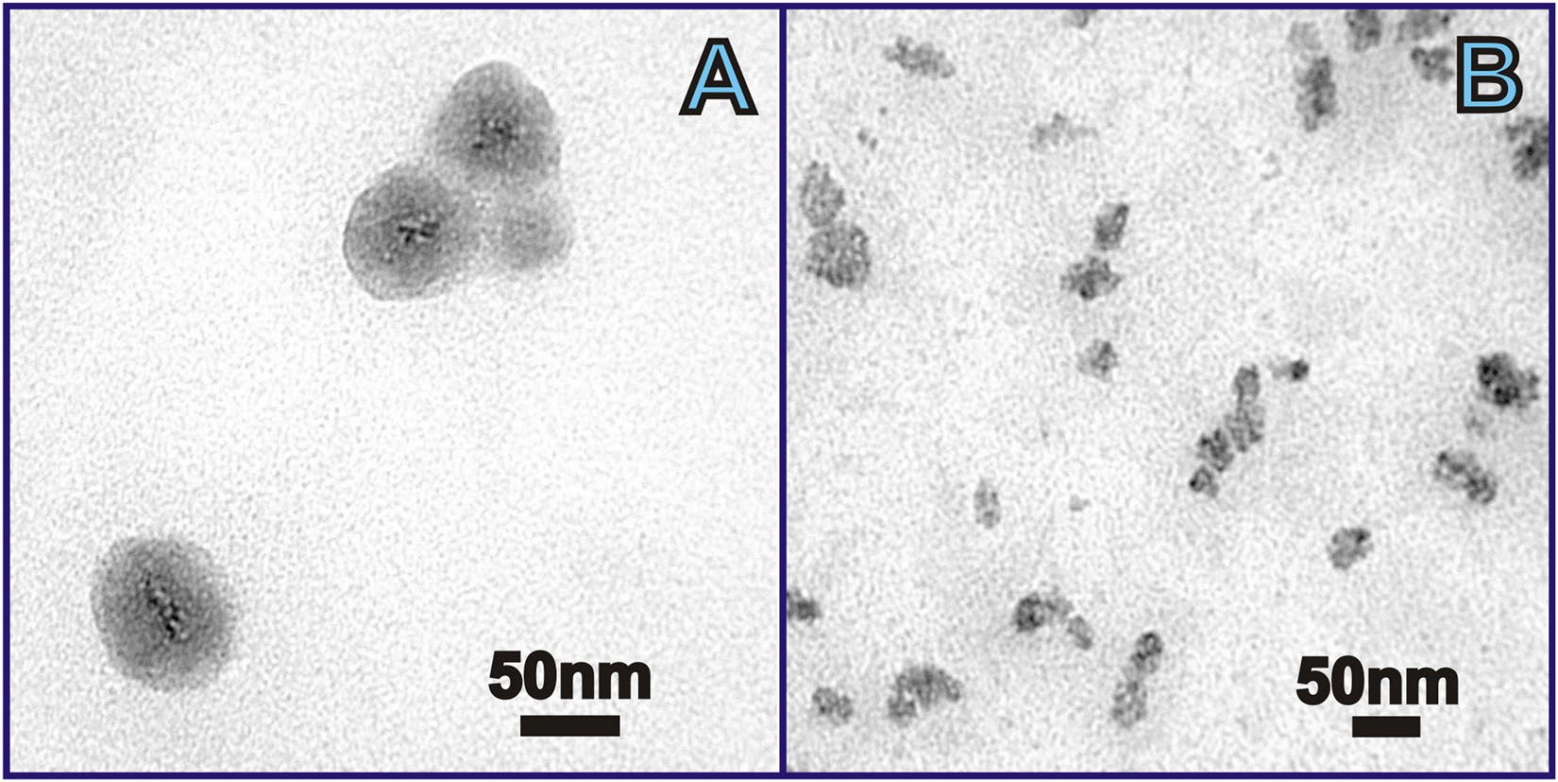
TEM image of dried PSS-coated [Gd(TTA)_3_·**1**] (**A**) and PSS-Na[Gd·**2**] (**B**) colloids.

More detailed information on the structure of the colloids has been obtained using TEM. TEM gives evidence of colloidal inhomogeneity. Small 2–5 nm sized grains, localized predominantly in the center of the particles, are detected as black dots scattered within 30–40 nm dark grey spheres (Fig. 3). According to the expected electronic density derived from the atomic composition, black and dark grey regions are assigned to [Gd(TTA)_3_·**1**] nanoparticles wrapped around with PSS chains, forming “papaya”-like nanostructures (Fig. 3a). The formation of these nanostructures is manifested by 150–170 nm sized aggregates in DLS measurements (Table 1SI). The aggregation of the PSS-stabilized ultra-small nanoparticles into the nanostructure is facilitated by counter-ion binding, since the PSS-based layer is enriched by sodium ions, which derive from the presence of sodium chloride in synthetic solution. The previously reported DLS results indicate that these nanostructures are very stable, which is evident from the stability of the size-values in time and under the dilution of the colloids^33^.

The size and shape of single particles in the dried state detected by TEM as well as the presence of agglomerates are in full agreement with AFM data.

The comparison of the AFM and TEM images (Figs 2 and 3) reveals similarity between PSS-[Gd(TTA)_3_·**1**] and PSS-Na[Gd·**2**] colloids in colloidal characteristics.

Moreover, Eu(III) and Tb(III) counterparts of studied colloids, namely PSS-[Eu(TTA)_3_·**1**] and PSS-Na[Tb·**2**], which are mandatory for photophysical investigations (see below), are fully isostructural with Gd(III) colloids. No effect of the nature of lanthanide ion on colloids parameters of the corresponding PSS-stabilized colloids was detected. This enables to assume that the colloidal parameters (e.g. size, shape, aggregation behavior) of PSS-[Gd(Eu)(TTA)_3_·**1**] and PSS-Na[Gd(Tb)·**2**] are resemble to each other. Thus, the differences in functional parameters can be ascribed exclusively to the nature of the Gd(III) chelates.

### Determination of hydration numbers of Gd(III) chelates in the form of colloids

Predominant localization of Ln(III) centers at the surface of each “papaya” seed inside of [Gd(Eu)(TTA)_3_·**1**] and Na[Gd(Tb)·**2**] nanoparticles is common for 2–4 nm sized nanoparticles^34,35^. Compared to bulk complexes, surface complexes are generally more labile and, hence, gadolinium cations are more hydrated, due to ligand exchange and dechelation processes taking place on the water-“papaya” seed interface. Thus, the effective hydration number of Gd(III) centers may differ from assumptions above (q = 1 in case of [Gd(TTA)_3_·**1**] and q = 4 in case of Na[Gd·**2**]-based colloids) and should be determined in conditions of the aqueous colloids. Horrocks method, based on the luminescence decay lifetimes studies of Ln(III) chelates in H_2_O and D_2_O may be used for this purpose as a reliable tool with extrapolation to the form of colloids^1,36–39^.

Gadolinium chelates are known to have high energy gap between triplet state of antenna-ligand as a photon supplier, and Gd(III) excited state level. This makes gadolinium chelates difficult to excite and, therefore, excludes the possibility of aquation studies of Gd(III) coordination center directly. For this purpose, non-luminescent Gd(III) was replaced with similarly coordinating red (614 nm) emissive Eu(III) in [Gd(TTA)_3_·**1**] complex and green (main emission band at 545 nm) emissive Tb(III) in Na[Gd·**2]**, respectively. Bright luminescence has been found for the aqueous colloids, based on [Eu(TTA)_3_·**1**] and Na[Tb·**2]** (Figure 2SI, Figure 3SI), enabling excited state lifetime measurements.

A single exponential decay of luminescence intensity after excitation is usually observed for each luminescent species. Each single exponent refers to one luminescent complex form or luminescent ligand. Multicomponent decay reflects co-existence of several complex forms (which are luminescent) in the sample. Radiative processes in lanthanide chelates compete with radiationless energy transfer through coupling of Ln(III) emissive states to the O-H vibrational levels of coordinated H_2_O molecules, substantially shortening the lifetime *τ* and lowering the emission intensity *I*_*lum*_ of lanthanide chelates. To investigate the role of O-H oscillators as de-excitation pathway, we synthesized PSS-[Ln-**L**] samples in H_2_O (O-H de-excitation path active) and D_2_O (O-H de-excitation path inactive), in parallel, to replace the O-H oscillators with O-D. Replacement of O-H to O-D oscillators occurring in pure D_2_O colloid dispersions causes the vibronic de-excitation pathway to become exceedingly inefficient.

**Figure 4:**
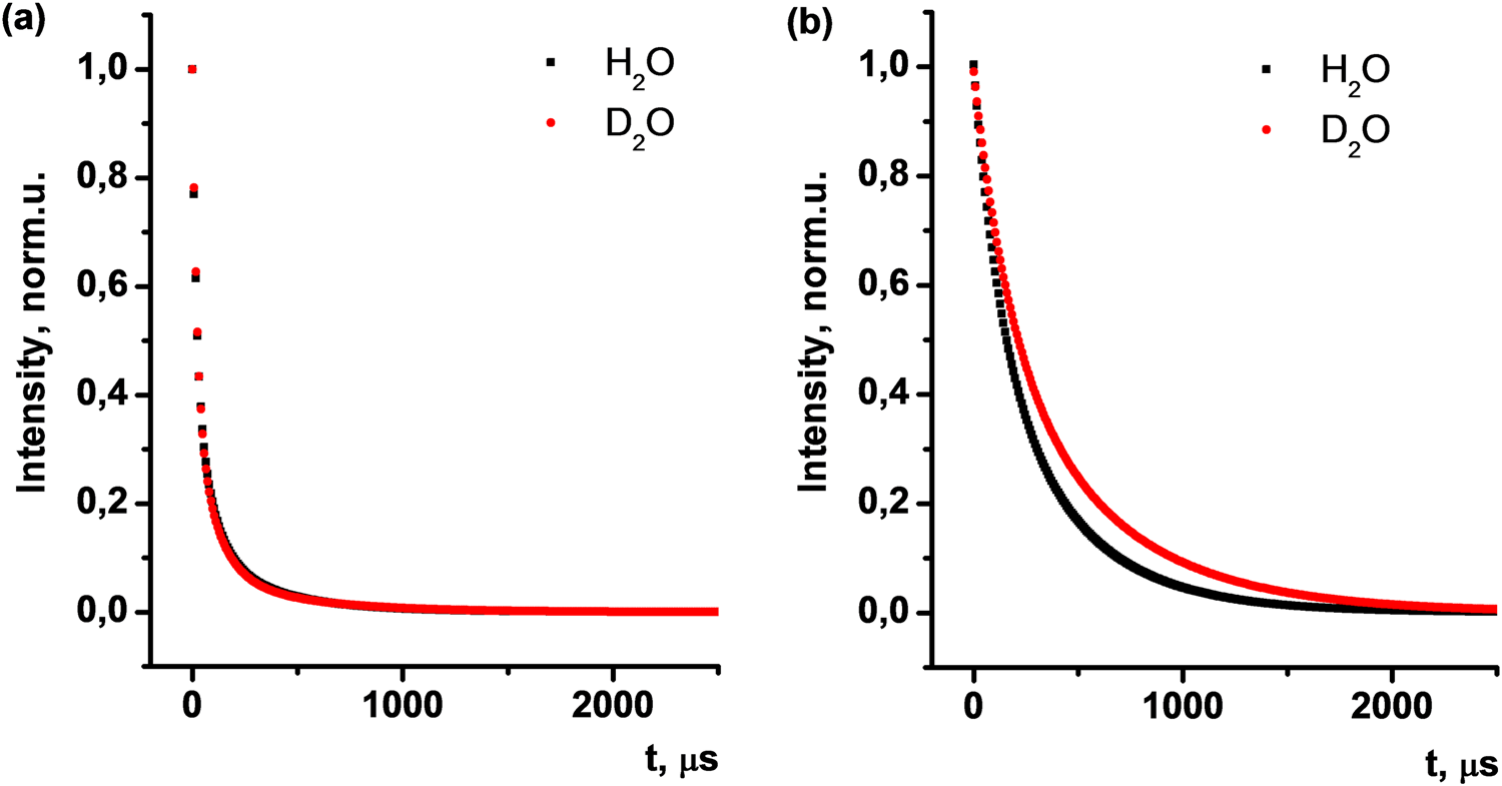
Excited state lifetime decay kinetics in PSS-Na[Tb·**2**] (**a**) and PSS-[Eu(TTA)_3_·**1**] (**b**) colloids in H_2_O (black) and D_2_O (red).

**Figure 5:**
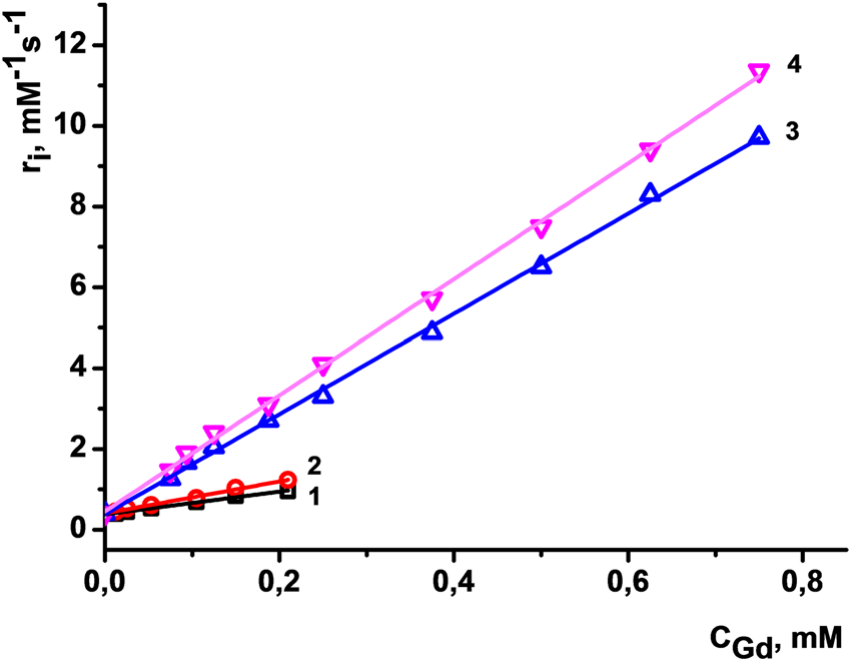
Longitudinal (1, 3) and transverse (2, 4) relaxation rates (1/T) versus Gd(III) concentration of PSS-[Gd(TTA)_3_ **1**] (1, 2) and PSS-Na[Gd 2] (3, 4) colloids measured at 0.47 T and 25 °C. Straight lines are linear fitting of the experimental data (statistical treatments are exemplified at Figure 8SI, Figure 9SI and Table 2SI, Table 3SI).

Excited state lifetime decay kinetics studies of Na[Tb·**2]** based colloids have been performed in H_2_O and D_2_O (Fig. 4). Experimentally obtained decay curves are best fitted with three exponential deconvolution (Pearson’s coefficient (R) 0.9999 for H_2_O and 0.9996 for D_2_O, respectively) (Figure 4SI, Figure 5SI). This fact points to the presence of three luminescent species in PSS-Na[Tb·**2**] aqueous colloids, characterized with lifetimes τ_1_ = 16.9 μs, τ_2_ = 80.6 μs and τ_3_ = 330.8 μs (Table 1). In case of PSS-Na[Tb·**2**] colloids in D_2_O the lifetimes are significantly increased, giving τ_1_ = 20.7 μs, τ_2_ = 97.2 μs and τ_3_ = 419.2 μs, which is expected as a consequence of the exclusion of the O-H de-excitation pathway.

**Table 1:** Excited state lifetimes (τ, μs) and Pearson’s coefficient (R) as a result of deconvolution of experimental decay curves in PSS-Na[Tb·**2**] and PSS-[Eu(TTA)_3_·**1**] aqueous colloids.

		τ_1_, μs	τ_2_, μs	τ_3_, μs	Pearson’s coefficient (R)
PSS-Na[Tb·**2**]	H_2_O	16.9	80.6	330.8	0.9999
	D_2_O	20.7	97.2	419.2	0.9996
PSS-[Eu(TTA)_3_·**1**]	H_2_O	123.5	398.2	—	0.9994
	D_2_O	165.3	545.3	—	0.9999

In contrast to organic molecules, the excited state lifetime for lanthanides lies typically in the milliseconds range. Therefore, the long τ_3_ component of the experimental decay can be unambiguously attributed to the lifetime of Tb(III) excited state in the bis-chelated form. The presence of τ_2_ may be referred to mono-chelated Na[Tb·**2**], which should be minor complex form, compared to the bis-chelated one. Taking into account previously reported τ values for similar macrocyclic 1,3-diketones^40^, short τ_1_ indicates some minor contribution of the free ligand **2** to the luminescence of the nanoparticles.

**Figure 6:**
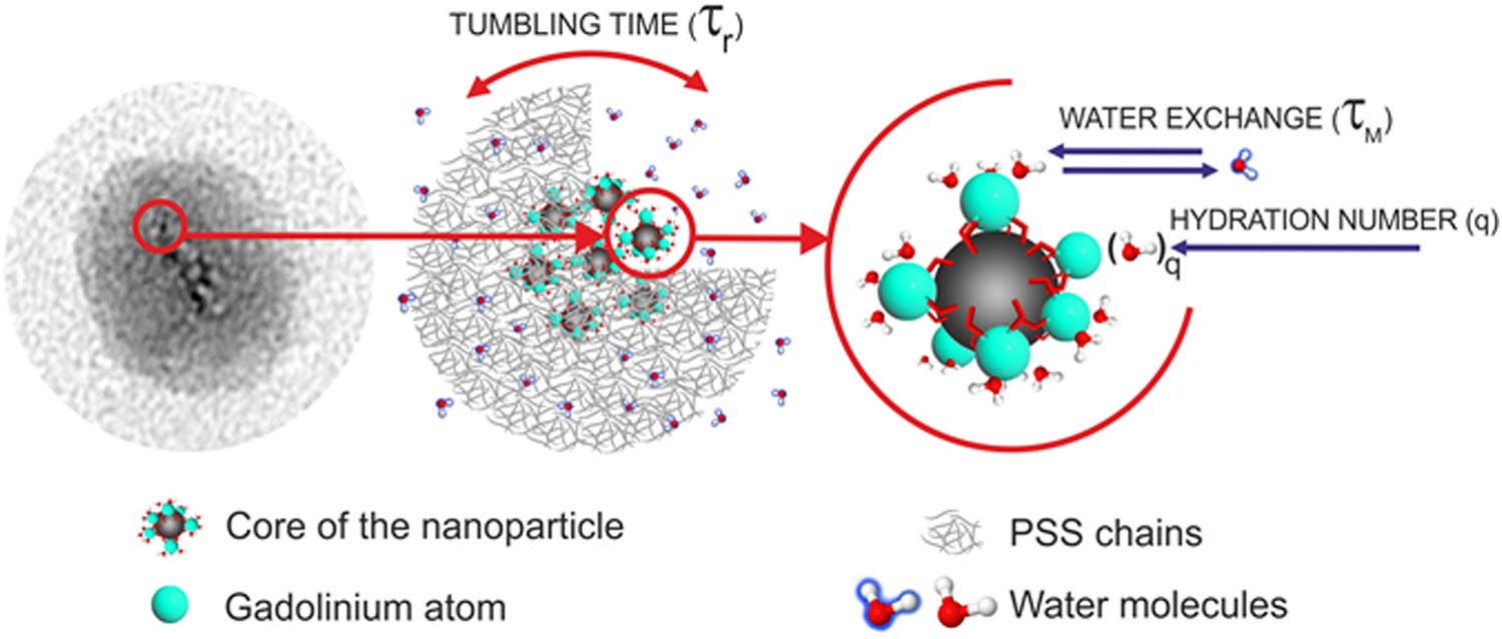
Schematic illustration of the nanoparticular core built from the Gd(III) complexes.

Tri-exponential fitting for PSS-[Eu(TTA)_3_·**1**] colloids in H_2_O and D_2_O (Fig. 4b) give nearly the same τ values with no gain in Pearson’s coefficient. Thus, deconvolution of experimental decay curves in this system results in bi-exponential fitting with a high R. Relatively long lifetimes τ_1_ = 123.5 μs and τ_2_ = 398.2 μs are evidence of bis- and tris-chelated europium(III) ions within the core of PSS-coated colloids (Figure 6SI, Figure 7SI). In contrast to PSS-Na[Tb·**2**] colloids, we found no contribution of ligand excited state lifetime in PSS-[Eu(TTA)_3_·**1**] colloids, which reflects the absence or the negligible contribution of non-bound to europium(III) ligands of TTA and **1**.

The comparison of the excited state lifetimes of Ln(III) in certain complexes in H_2_O and D_2_O solutions allows calculating the number of coordinated water molecules via Horrocks equation (Equation 1) with the uncertainty in *q* (number of water molecules) of ±0.5^36,41^.

1 $${q}_{Ln}={A}_{Ln}({{\tau }^{-1}}_{{H}_{2}O}-{{\tau }^{-1}}_{{D}_{2}O})$$

In Equation 2, q_Ln_ represents the number of water molecules coordinated to the particular lanthanide, A_Ln_ is a constant specific for each Ln(III), defined empirically for Ln(III) chelates with known q, *τ* ^−1^ are the reverse values of excited state lifetimes of Ln(III) in H_2_O or D_2_O.

The significant increase of excited state lifetimes of luminescent lanthanides(III) located within the core of colloids upon change of medium from H_2_O to D_2_O (Table 2) proves that water molecules interact with Ln(III) centers located at the surface of nanosized heterogenic “papaya”-like seeds occupying the free coordination places in the nearest environment of Ln(III).

**Table 2:** Excited state lifetime (τ, ms), hydration number (q), spin-lattice (r_1_, mM^−1^s^−1^), and spin-spin (r_2,_ mM^−1^s^−1^) relaxivity values of PSS-[Gd(Eu)(TTA)_3_·**1**], PSS-Na[Gd(Tb)·**2**] colloids.

	τ _H2O_, ms	τ _D2O_, ms	q (±0.5)	r_1,_ mM^−1^s^−1^	r_2_, mM^−1^s^−1^
PSS-[Gd(Eu)(TTA)_3_·**1**]	0.398 ± 0.001	0.545 ± 0.001	0.71	2.82 ± 0.11	3.95 ± 0.13
PSS-Na[Gd(Tb)·**2**]	0.331 ± 0.001	0.419 ± 0.003	2.65	12.41 ± 0.16	14.36 ± 0.15

Using constant values of A_Ln_ (A_Eu_ = 1.05; A_Tb_ = 4.2) and measured *τ* in milliseconds^1,36^, the hydration numbers were deduced for both [Gd(TTA)_3_·**1**] and Na[Gd·**2**] complexes in PSS-[Eu(TTA)_3_·**1**] and PSS-Na[Tb·**2**] colloids using Equation 2. The calculated hydration number (*q*) of Na[Tb·**2**] in PSS-Na[Tb·**2]** colloids (q≈3) is higher than the same factor for [Eu(TTA)_3_·**1**] in isomorphic colloids of PSS-[Eu(TTA)_3_·**1**] (q≈1).

It is worth noting that measurements and calculations of *q* according to *Horrocks* were previously applied to the lanthanide complexes in solution, crystalline solids and protein-metal complexes^40^. Herein, we have expanded the applicability of the quantification of the hydration number to lanthanide centers distributed within the surface of nanoparticulate 2–5 nm cores of core-shell heterogeneous systems. The obtained values point to insignificant dechelation of Tb(III) lantanide ions in the colloids. Moreover, the obtained q-value for PSS-Na [Gd **2**] (q≈3) is somewhat lower than the anticipated hydration value (q = 4). This fact may be explained by the steric hindrance of non-coordinating 1,3-diketone groups of ligand **2**.

Application of Horrocks theory demonstrates the possibility of producing two different systems, very similar in morphology, but very different in their hydration state. We then measured the water protons nuclear magnetic relaxation efficiency for high hydrated (q≈3 in PSS-Na[Gd·**2**]) and low hydrated (q≈1 in PSS-[Gd(TTA)_3_·**1]**) Gd(III) 1,3-diketonates, to evaluate the role of the number of water molecules in the first coordination sphere of Gd(III) chelate in relaxivity of Gd(III) complex-based nanoparticles.

### Hydration number - Nuclear magnetic relaxivity correlation

Both Gd(III) complexes and Gd(III)-based nanoparticles refer to the so-called T_1_-probes due to low r_2_/r_1_ ratio. The higher longitudinal relaxivity, the brighter is the region of image where the probe is accumulated, and the lower is concentration of the CA needed for effective contrasting. For this reason, the top challenge is to improve the sensitivity of T_1_-probes. The relaxivity of mononuclear or nanoparticulate Gd(III) complexes at a fixed external magnetic field, *r*, is defined as the water protons relaxation enhancement in the presence of paramagnetic species, normalized to their concentration. According to the Solomon–Bloembergen–Morgan (SBM) theory^42^, which is derived for homogenous solutions of paramagnetic complexes, relaxivity (*r*_1_) is mainly contributed by dipole-dipole interactions of inner-sphere coordinated water molecules with paramagnetic ion at low frequencies (20–60 MHz)^35^.

The Equation 2 illustrates the dependence of r_1_ on inner-sphere hydration number (q)^43^, although this equation cannot be directly applied for Gd(III) complexes in the nanoparticulate form^42^.

2 $${r}_{{1}}^{IS}=(q/[{H}_{{2}}O])/({T}_{{1}m}+{\tau }_{m}),$$

3 $$1/{T}_{1M}=K/{r}_{H}^{6}(3{\tau }_{C1}/(1+{\omega }_{H}^{2}{\tau }_{C1}^{2})+7{\tau }_{C2}/(1+{\omega }_{S}^{2}{\tau }_{C2}^{2}))$$

where r_1_^IS^ is the inner-sphere water relaxivity; T_1m_ refers to the T_1_ of the coordinated water proton according to Equation 2, and is the function of proton Larmor frequency (ω) and correlation time of the inner-sphere water relaxation (τ_c_) in accordance with Equation 3.

Taking into account the well-known SBM theory equations^42^ and the negligible contribution of *τ*_*s*_^−1^ for Gd(III) chelates, *r*_1_ is governed by the rotational correlation time or molecular tumbling (τ_r_), the proton residence time (τ_m_), and the number of water molecules in the first coordination sphere of Gd(III)^43^.

The presence of chelating ligand in the inner-sphere of Gd(III) is well-known to affect the water exchange rate. Moreover, the nature of donor atoms can significantly tune the water exchange kinetics^44,45^. Taking into account that Gd(III) ions in both the complexes are chelated by oxygen ions, mainly deriving from 1,3-diketonate chelates, the difference in *τ*_*m*_ is not so important in the relaxivity of [Gd(TTA)_3_·**1**] and Na[Gd·**2**]. It is also worth noting that *τ*_*r*_^−1^ gives typically the predominant contribution to *τ*_*c*_^−1^ for mononuclear Gd(III) complexes^42^.

We have previously reported^31^ that τ_r_ for PSS-Na[Gd·**2**] is about 2 ns, which is relevant to slow tumbling regime. As it has been above mentioned the colloid characteristics of PSS-[Gd(TTA)_3_·**1**] and PSS-Na[Gd·**2**] are rather close to each other, which points to similar slow tumbling regime for the Gd(III) centers in both the colloids. The similar morphology of the both colloids, in turn, enables to assume the similarity in (T_1m_ + τ_m_) values for PSS-[Gd(TTA)_3_·**1**] and PSS-Na[Gd·**2**]. It is worth noting that the slow tumbling regime is not the only factor guiding the relaxivity, since the decreased contribution of *τ*_*r*_^−1^ *to τ*_*c*_^−1^ results in the enhanced impact of *τ*_*m*_^−1^ and *τ*_*s*_^−1^ on the relaxation mechanism. The optimal combination of *τ*_*m*_ and *τ*_*r*_ values is highlighted as the most important prerequisite for high relaxivity^40^. Taking into account that the relaxivity values measured for both the colloids are lower than the best examples with the relaxivity values higher than 50 mM^−1^∙s^−1^, the *τ*_*m*_ values for the two colloids are far from the optimal ones. Nevertheless, the impact of the inner-sphere hydration on the relaxivity in Gd(III)-based aqueous colloids can be revealed on a qualitative level by the comparison of the longitudinal and transverse relaxivity of the Gd(III)-based PSS-stabilized colloids with different hydration numbers. It is worth noting that another factors, such as accessibility of Gd(III) centers should be taken into account. As it has been mentioned above, the deviation in the size of PSS-[Gd(Eu)(TTA)_3_·**1**] and PSS-Na[Gd(Tb)·**2**] is negligible. Nevertheless, difference in volume of complexes can be a reason for different accessibility of Gd(III) centers for hydration. The molecular modeling calculations enable to evaluate the volume ratio of complexes [Gd(Eu)(TTA)_3_·**1**] and Na[Gd(Tb)·**2**]. The volume ratio, derived as a sum of chelates’ ionic radii, is 0.85, which can be a reason for some deviation, but not for a dramatic difference.

Figure 5 illustrates the difference in the r_1_ values obtained from the linear dependencies of 1/T_1_ and 1/T_2_ values on Gd(III) concentration for aqueous colloids PSS-[Gd(TTA)_3_·**1**] and PSS-Na[Gd·**2**]. The dramatic difference in the r_1_ values confirms the impact of the inner-sphere hydration on a qualitative level. Moreover, the ratio of the r_1_ values for the colloids PSS-[Gd(TTA)_3_·**1**] and PSS-Na[Gd·**2**] (2.82 to 12.41) is close to the ratio of their q according Equation 4 (0.71 to 2.65).

Thus, having one water molecule in the first coordination sphere of Gd(III), PSS-[Gd(TTA)_3_·**1**] colloids show poor spin-lattice (r_1_ = 2.82 mM^−1^s^−1^) and spin-spin (r_2_ = 3.95 mM^−1^s^−1^) relaxivities (Table 2). One water molecule is not sufficient to induce effective relaxation via both spin-spin and spin-lattice mechanisms for Gd chelate-based colloidal architectures.

The relaxation rates increase dramatically upon growth of the number of water molecules in the nearest neighborhood to Gd(III) paramagnetic center. Longitudinal (spin-lattice) and transverse (spin-spin) rates reach 12.41 mM^−1^s^−1^ and 14.36 mM^−1^s^−1^ values for PSS-Na[Gd·**2**], respectively. These values are comparable with the relaxation rates of Gd^3+^ aqua ion (r_1_ = 14 mM^−1^s^−1^) and are three times higher than commercial MRI contrast agents as Magnevist and Omniscan (r_1_ = 4.1 mM^−1^s^−1^ and 4.3 mM^−1^s^−1^, respectively^46^). The existence of three water molecules in PSS-Na[Gd·**2**] colloids along with slow tumbling regime for Gd(III) centers determines the higher relaxation ability of the colloids and, hence, much higher sensitivity in contrasting. At the same time, nanoparticulate morphology represented herein is superior to conventional molecular contrast agents (CA) in terms of Gd^3+^ leakage and hence toxicity towards cells and organs^18^. The obtained results reveal an impact of the macrocyclic platform bearing 1,3-diketonate chelating substituents, which both provide tight binding of the lanthanide ions via two chelating groups and prevent the inner sphere coordination of the third one.

Schematically, the composition of each grain may be illustrated as a conglomeration of gadolinium chelates, coated with polyelectrolyte chains (Fig. 6). The latter ones are permeable for the water molecules, which may penetrate the polyelectrolyte layer and interact with the gadolinium ions on the surface of “papaya” seeds, enhancing the relaxation of water protons. It is also worth noting that the diffusion rate of water molecules is somehow restricted by the polyelectrolyte-based exterior layer, which can affect the efficiency of the inner sphere water exchange shown in Fig. 6.

The applicability of PSS-Na[Gd·**2**] colloids as MRI contrast agents requires their stability under heating up to 37 °C, which is the typical temperature for living systems. Both luminescent (for PSS-Na[Tb·**2**] colloids) and magnetic (for PSS-Na[Gd·**2**] colloids) relaxation measurements were performed at 37 °C and compared with those performed at room temperature (Figure 10SI, Table 4SI). The data indicate very small deviation between the values determined at different temperatures, which points to the thermal stability of the investigated colloidal systems and, hence, their applicability in living systems.

## Conclusion

The present work introduces two types of the Gd(III)-based colloids with very similar core-shell morphology and size, where the hard cores are based on the complexes of different structure. In particular, complex [Gd(TTA)_3_·**1**], where the lanthanide ions are coordinated with three 1,3-diketonate of TTA^−^ and the phosphine oxide derivative and Na[Gd·**2**] complex, where the coordination of the lanthanide ion undergoes via two chelating 1,3-diketonates embedded to macrocyclic cavitand backbone. The luminescence of both PSS-[Eu(TTA)_3_·**1**] and PSS-Na[Tb·**2**] colloids enables to measure the inner-sphere hydration numbers of the lanthanide ions (q). The estimated q values are in quantitative relationship with the measured relaxivity values for PSS-[Gd(TTA)_3_·**1**] and PSS-Na[Gd·**2**] colloids. This tendency reveals water insoluble Gd(III) complexes with high inner sphere hydration as promising candidates for aqueous Gd(III)-based colloids. Heterogenic nature of the synthesized colloids, the water insolubility of the core material, the nanosized structure and the tight 1,3-diketone chelation eliminate drawbacks of molecular Gd^3+^ contrast agents along with preserving the effective Gd-to-water interaction. The regularities obtained in this work are fundamental in understanding the efficiency of MRI probes in the fast growing field of nanoparticulate contrast agents.

## Experimental

### Reagents and materials

Gadolinium nitrate Gd(NO_3_)_3_·6H_2_O (99.9%) (Alfa Aesar), terbium nitrate hydrate (Tb(NO_3_)_3_∙5H_2_O) (Alfa Aesar) triethylamine (TEA) (Acros Organics), poly(sodium 4-styrenesulfonate) (PSS) (MWaverage = 70000) (Acros Organics), sodium chloride (Sigma-Aldrich), were used as commercially received without further purification. N,N-Dimethylformamide (DMF) (Acros Organics) was twice distilled over P_2_O_5_.

Syntheses of Z-2-(2-hydroxy-4-methyl-5-chlorphenyl)-2-phenylethenyl-bis(3,4-dimethoxyphenyl) phosphine oxide (**1**) and 5,11,17,23-tetrakis[(acetylaceton-3-yl)methyl)]-25,26,27,28-tetrahydroxy-calix[4]arene were reported previously^27,28^.

### Synthesis

Polyelectrolyte nanoparticles have been synthesized using well-known “layer-by-layer” technique, where polyelectrolyte molecules adsorbed on the surface of nanosized templates. Hard nanosized templates were obtained via precipitation of water non-soluble gadolinium(III) complexes with corresponding macrocyclic ligands under intensive stirring (2200 rpm) from organic to negatively charged PSS-containing water (1 g·L^−1^ in 0.5 М solution of NaCl, рН 6.0).

Organic to water phase’s volume ratio has been maintained at 1 to 5. The resulted colloidal solution has been sonicated for 30 minutes (sonication bath temperature was controlled to be 20 ± 2 °С). Excess amounts of PSS were separated from colloids via centrifugation (10000 rpm, 15 min) and supernatant drain. One layered PSS-coated aqueous colloids were obtained via redispergation (using sonication) in bidistilled water (equal to supernatant volume).

## Methods

*TEM images* were obtained with Hitachi HT7700, Japan. The images were acquired at an accelerating voltage of 100 kV. Samples were dispersed on 300 mesh copper grid with continuous carbon-formvar support film.

*Atomic force microscope* (MultiMode V, USA) was used to reveal the morphology of the samples. The 250–350 kHz cantilevers (Veeco, USA) with silicone tips (tip curvature radius is of 10–13 nm) were used in all measurements. The microscopic images were obtained with 512 × 512 resolution. The scanning rate was 1 Hz. The antivibrational system (SG0508) was used to eliminate external distortions. Droplets of aqueous dispersions of the samples were carefully placed on mica surface with the roughness no more than 1–5 nm. The AFM imaging was performed after water evaporation.

*Dynamic light scattering (DLS)* measurements were performed by means of the Malvern Mastersize 2000 particle analyzer. A He-Ne laser operating at 633 nm wavelength and emitting vertically polarized light was used as a light source. Measured autocorrelation functions were analyzed by Malvern DTS software and the second-order cumulant expansion methods. *Zeta potential “Nano-ZS”* (MALVERN) using laser Doppler velocimetry and phase analysis light scattering was used for zeta potential measurements.

*Longitudinal (T*_1_) *and transverse relaxation times (T*_2_) of water protons in aqueous solutions of newly synthesized Gd compounds were performed at 19.65 MHz proton resonance frequency at room temperature. The relative measurement deviation for transverse and longitudinal relaxation times does not exceed 3%. Transverse r_2_ = 1/(*T*_2_C_Gd_), mM^−1^s^−1^ and longitudinal r_1_ = 1/(*T*_1_C_Gd_), mM^−1^s^−1^ relaxivities were calculated from the measured relaxation times *T*_2_ and *T*_1_ respectively, where C_Gd_ designates gadolinium concentrations. The samples were sonicated using ultrasound bath for 60 minutes prior to relaxometric measurements. Bruker “Minispec mq20” NMR analyzer were employed to measure *T*_1_ and *T*_2_ of water molecule protons in studied solutions at 0.47 T. Inversion-recovery and Carr-Purcell-Meiboom-Gill (CPMG) pulse sequences were used for T_1_ and T_2_ relaxation times accordingly with 20 points data collected for fitting.

*The steady-state luminescence spectra* were recorded using spectrometer based on MDR-2 and MDR-12 monochromators was used in our experiments. The excitation was done by the required band of the light produced from the broad spectrum of a 1 kW xenon lamp to obtain the luminescence and excitation spectra. A pulse N2 laser LGI 21 (carrying wave length of 337 nm, pulse duration of 12 ns) was used to obtain the time-resolved data. The optical signal was detected on a FEU-79 photoelectron multiplier with abroad band amplifier.

### Data availability statement

All data generated or analyzed during this study are included in this published article (and its Supplementary Information files).

## Electronic supplementary material

Supplementary Information
