# Supplementary material for: Hydration number: crucial role in nuclear magnetic relaxivity of Gd(III) chelate-based nanoparticles
Source: Sci Rep. 2017 Oct 25;7:14010. doi: 10.1038/s41598-017-14409-6 (PMC5656664; doi:10.1038/s41598-017-14409-6)
Supplement: Supplementary file 1 — Supplementary Information [file 41598_2017_14409_MOESM1_ESM.doc]

**Hydration number: crucial role in nuclear magnetic relaxivity of Gd(III) chelate-based nanoparticles.**

Rustem Zairov, Gulshat Khakimullina, Sergey Podyachev, Irek Nizameev, Georgy Safiullin, Rustem Amirov, Alberto Vomiero, Asiya Mustafina

**Supplementary information.**


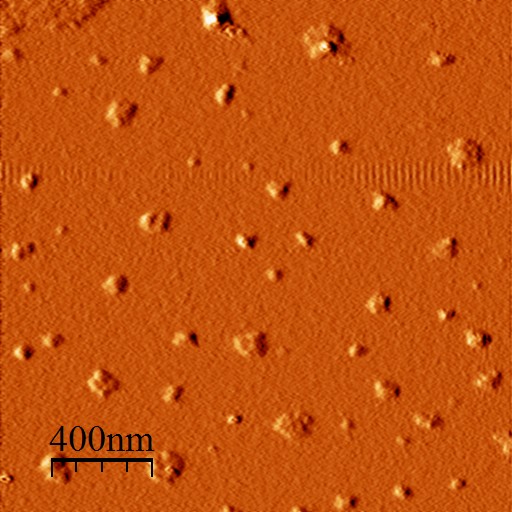

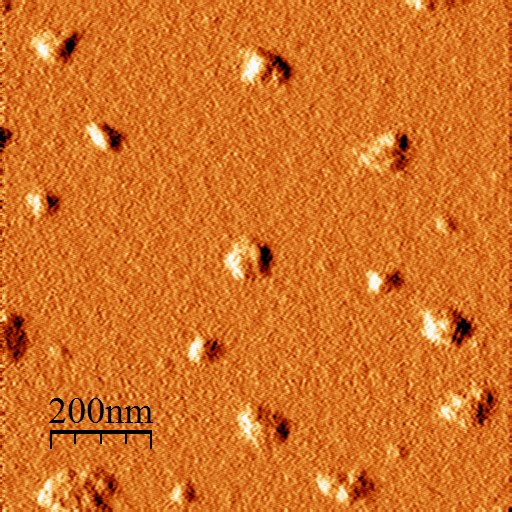


*Fig. 1SI AFM images of dried PSS-coated [Gd·****2****] colloids at different magnification.*

*Fig. 2SI Luminescence spectrum of PSS-[Eu(TTA)_3_·****1****] colloids. λ_ex_=370 nm.*

*Fig. 3SI Luminescence spectrum of PSS-Na[Tb·****2****] colloids. λ_ex_=320 nm.*

y=-480,8+197,3e^(-(x+19,9)/80,6)^+707,8e^(-(x+19,9)/16,9)^+55,0e^(-(x+19,9)/330,8)^ R=0,99988

*Fig. 4SI Decay kinetics in PSS-Na[Tb·****2****] colloids in H_2_O.*

y=-480,9+286,9e^(-(x+33,4)/97,2)^+54,5e^(-(x+33,4)/419,2)^+1888,7e^(-(x+33,4)/20,7)^ R=0,9996

*Fig. 5SI Decay kinetics in PSS-Na[Tb·****2****] colloids in D_2_O.*

y=0,0008+0,5e^(-(x+8,4)/123,5)^+54,5e^(-(x+8,4)/398,2)^ R=0,99941

*Fig. 6SI Decay kinetics in PSS-[Eu(TTA)_3_·****1****] colloids in H_2_O.*

y=0,0008+0,4e^(-(x+9,4)/165,3)^+0,6e^(-(x+9,4)/545,3)^ R=0,99991

*Fig. 7SI Decay kinetics in PSS-[Eu(TTA)_3_·****1****] colloids in D_2_O.*

*Table 1SI. Hydrodynamic diameter (d, nm), polydispersity indices (PDI) and electrokinetic potential (ζ, mV) of PSS-coated polyelectrolyte nanoparticles PSS-[Gd(TTA)_3_·****1****], PSS-Na[Gd·****2****].*

|  | d, nm | PDI | ζ, mV |
| --- | --- | --- | --- |
| PSS-[Gd(TTA)_3_·**1**] | 168±2 | 0.268 | -40.5±1.2 |
| PSS-Na[Gd·**2**] | 155±4 | 0.220 | -42.4±0.2 |

*Table 2SI. Longitudinal relaxation time (T_1_, ms) and rate (1/T_1_, s^-1^), and transverse relaxation time (T_2_*, *ms) and rate (1/T_2_, s^-1^) of PSS-coated nanoparticles on the basis of [Gd(TTA)_3_·****1****].*

| C_PSS-[Gd(TTA)3·_**_1_**], mM | *T_1_*, ms | 1/T_1_, s^-1^ | *T_2_*, ms | 1/T_2_, s^-1^ |
| --- | --- | --- | --- | --- |
| 0.21 | 1050 | 0.952 | 813 | 1.23 |
| 0.15 | 1202 | 0.832 | 971 | 1.03 |
| 0.105 | 1466 | 0.682 | 1282 | 0.78 |
| 0.053 | 1905 | 0.525 | 1639 | 0.61 |
| 0.0252 | 2268 | 0.441 | 1887 | 0.53 |
| 0.0126 | 2532 | 0.395 | 2222 | 0.45 |
| - | 2500 | 0.4 | 2500 | 0.4 |

*Table 3SI. Longitudinal relaxation time (T_1_, ms) and rate (1/T_1_, s^-1^), and transverse relaxation time (T_2_, ms) and rate (1/T_2_, s^-1^) of PSS-coated nanoparticles on the basis of Na[Gd·****2****].*

| C_PSS-Na[Gd·_**_2_**], mM | *T_1_*, ms | 1/T_1_, s^-1^ | *T_2_*, ms | 1/T_2_, s^-1^ |
| --- | --- | --- | --- | --- |
| 0.75 | 103 | 9.709 | 88 | 11.364 |
| 0.375 | 205 | 4.878 | 175 | 5.714 |
| 0.1875 | 369 | 2.71 | 322 | 3.106 |
| 0.125 | 487 | 2.053 | 415 | 2.41 |
| 0.09375 | 599 | 1.669 | 526 | 1.901 |
| 0.075 | 792 | 1.263 | 685 | 1.46 |
| - | 2500 | 0.4 | 2500 | 0.4 |

y=0,0004+0,0124x (1) R=0,9993

y=0,0005+0,0144x (2) R=0,9996

*Figure 8SI. Longitudinal (1/T_1_, 1) and transverse (1/T_2_, 2) relaxation rates versus Gd(III) concentration of PSS-Na[Gd* ***2****] colloids measured at 0.47 T and related linear fittings.*

y=0,0004+0,0039x (1) R=0,9931

y=0,0004+0,0028x (2) R=0,9963

*Figure 9SI. Longitudinal (1/T_1_, 1) and transverse (1/T_2_, 2) relaxation rates versus Gd(III) concentration of PSS-[Gd(TTA)_3_* ***1****] colloids measured at 0.47 T and related linear fittings.*


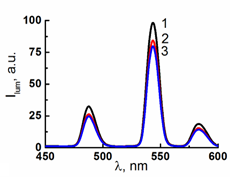


*Figure 10SI. Luminescence spectra of PSS-Na[Tb·****2****] colloids at 25 ^о^C (1), 37 ^о^C (2), exposure at 37 ^о^C for 1 hour. λ_ex_=320 nm.*

*Table 4 SI. Longitudinal relaxation time (T_1_, ms) and transverse relaxation time (T_2_, ms) of PSS-coated nanoparticles on the basis of Na[Gd·****2****] in H_2_O at 25 ^о^C and 37 ^о^C (C_Gd_=0.75 mM).*

| Temperature, ^о^C | Exposure, min | T_1_, ms | T_2_, ms |
| --- | --- | --- | --- |
| 25 |  | 103 | 88 |
| 37 | 10 | 106 | 90 |
| 37 | 60 | 109 | 94 |
